# A kinome-centered CRISPR-Cas9 screen identifies activated BRAF to modulate enzalutamide resistance with potential therapeutic implications in *BRAF*-mutated prostate cancer

**DOI:** 10.1038/s41598-021-93107-w

**Published:** 2021-07-01

**Authors:** Sander A. L. Palit, Jeroen van Dorp, Daniel Vis, Cor Lieftink, Simon Linder, Roderick Beijersbergen, Andries M. Bergman, Wilbert Zwart, Michiel S. van der Heijden

**Affiliations:** 1 Division of Molecular Carcinogenesis, Netherlands Cancer Institute, Plesmanlaan 121, 1066 CX Amsterdam, The Netherlands; 2 Department of Medical Oncology, Netherlands Cancer Institute, Amsterdam, The Netherlands; 3 NKI Robotics and Screening Center and ScreeninC, Netherlands Cancer Institute, Amsterdam, The Netherlands; 4 Division of Oncogenomics, Netherlands Cancer Institute, Amsterdam, The Netherlands; 5 Oncode Institute, Netherlands Cancer Institute, Amsterdam, The Netherlands

**Keywords:** Cancer genetics, Cancer therapy, Oncogenes, Tumour biomarkers, Urological cancer, Biotechnology, Cancer, Cell biology, Genetics, Molecular biology, Medical research, Molecular medicine, Oncology, Pathogenesis

## Abstract

Resistance to drugs targeting the androgen receptor (AR) signaling axis remains an important challenge in the treatment of prostate cancer patients. Activation of alternative growth pathways is one mechanism used by cancer cells to proliferate despite treatment, conferring drug resistance. Through a kinome-centered CRISPR-Cas9 screen in CWR-R1 prostate cancer cells, we identified activated BRAF signaling as a determinant for enzalutamide resistance. Combined pharmaceutical targeting of AR and MAPK signaling resulted in strong synergistic inhibition of cell proliferation. The association between BRAF activation and enzalutamide resistance was confirmed in two metastatic prostate cancer patients harboring activating mutations in the *BRAF* gene, as both patients were unresponsive to enzalutamide. Our findings suggest that co-targeting of the MAPK and AR pathways may be effective in patients with an activated MAPK pathway, particularly in patients harboring oncogenic *BRAF* mutations. These results warrant further investigation of the response to AR inhibitors in *BRAF*-mutated prostate tumors in clinical settings.

## Introduction

Prostate cancer is the second most common cancer diagnosed in men, accounting for over 350,000 cancer-related deaths worldwide each year^1^. The androgen receptor (AR) pathway is a key driver in prostate tumorigenesis, regulating genes that drive prostate cancer cell proliferation^2^. In recent years, new compounds have been introduced clinically that target the AR signaling axis resulting in tumor regression. These include drugs such as abiraterone, which blocks biosynthesis of androgen precursor molecules, and enzalutamide, which functions through antagonistic binding of AR. Even though these AR-directed drugs have shown to be clinically effective^3,4^, evasion of AR blockade through adaptation inevitably leads to disease progression and eventually death^5–7^. Acquired resistance to enzalutamide has been the focus of intense research, and several mechanisms have been described. These resistance mechanisms include activation of other signaling pathways such as the PI3K pathway^8^, NF-κB signaling^9^ and glucocorticoid receptor (GR) overexpression^10,11^.

Primary resistance is commonly defined by unresponsiveness to treatment, characterized by clinical progression within the first 3 months after commencing systemic therapy^5^. Primary resistance to enzalutamide, even though relatively under-examined, occurs in about 10% to 20% of prostate cancer patients^3,12^. A better mechanistic understanding of primary resistance will allow for better patient stratification and improved therapeutic avenues.

Functional genetic screens using CRISPR-Cas9 are a powerful tool for the unbiased identification of genes that have a central role in a wide range of biological processes in various genetic and pharmacological backgrounds, including cancer^11,13,14^. For example, through a genome-wide CRISPR-Cas9 screen, TLE3 was identified as a novel modulator of enzalutamide sensitivity which, together with AR, regulates GR expression and drug response in prostate cancer^11^. Using a similar approach, we set out to identify kinases whose inhibition could potentiate enzalutamide efficacy in prostate cancer cells, with the aim to discover biomarkers for resistance and potential drug combinations that are able to overcome enzalutamide resistance. We found that inhibition of BRAF, or downstream MAPK components MEK and ERK, enhanced enzalutamide sensitivity in prostate cancer cells harboring a mutation in the activating kinase domain of the *BRAF* gene. Our findings suggest therapeutic potential for co-inhibition of the MAPK and AR pathways in *BRAF*-mutated prostate cancers.

## Results

### A kinome-centered dropout screen identifies BRAF as a modulator of enzalutamide sensitivity

**Figure 1:**
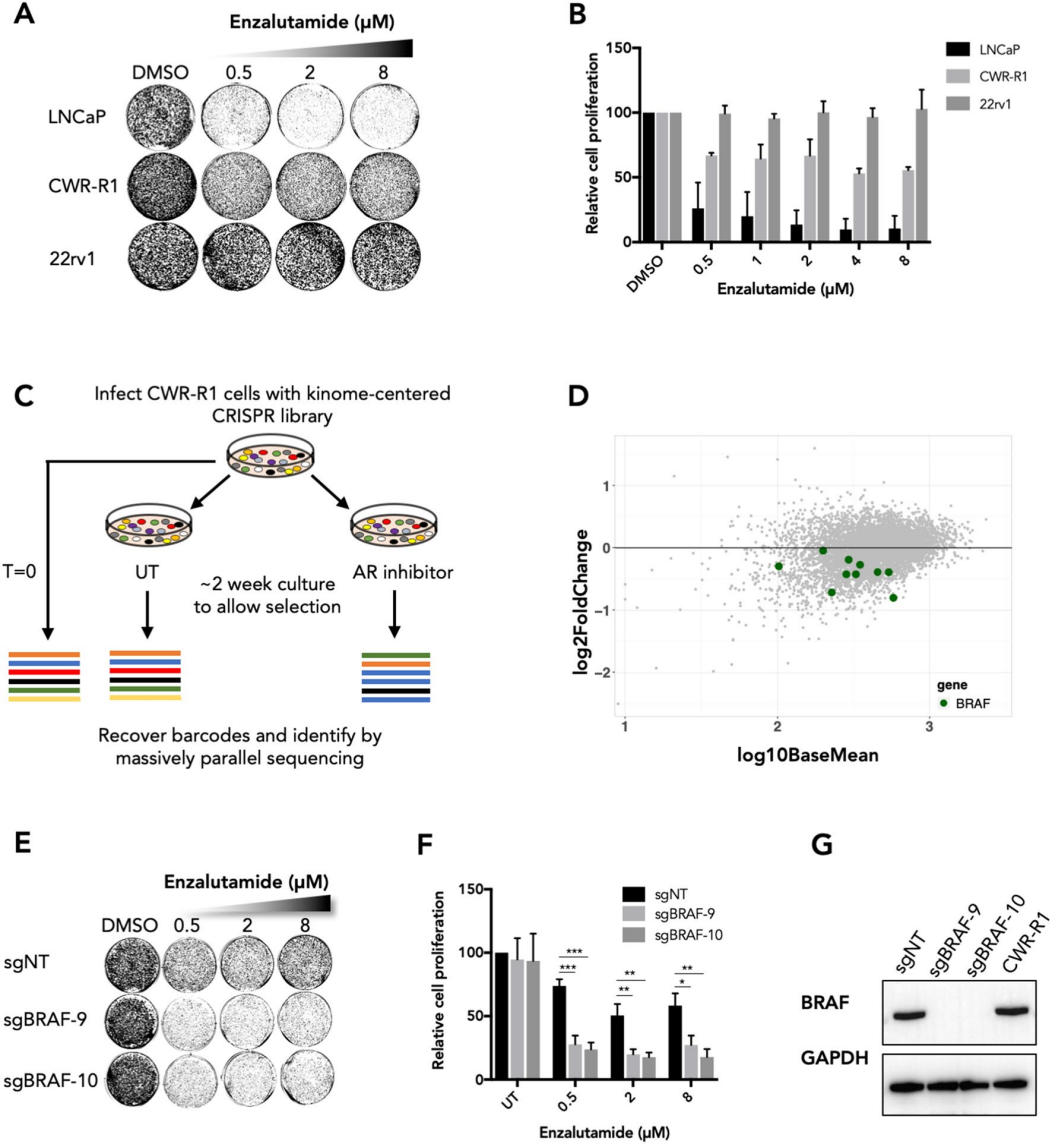
A kinome-centered CRISPR-Cas9 screen identifies *BRAF* as a modulator of enzalutamide sensitivity in CWR-R1 cells. (**A**). Enzalutamide sensitivity of prostate cancer cell lines LNCaP, CWR-R1 and 22rv1 in a long-term growth assay. (**B**) Quantified data of the results shown in (**A**). (**C**) Schematic representation of the kinome-centered CRISPR-Cas9 screen. (**D**) Representation of the relative abundance of the sgRNA barcode sequences of the screen. The y-axis shows the enrichment (relative abundance of enzalutamide treated/untreated) and the x-axis shows the average sequence reads of the untreated samples. (**E**) Long-term growth assay showing the enzalutamide response of CWR-R1 cells harboring sgRNAs targeting *BRAF*. Cells harboring a non-targeting sgRNA were used as a control. (**F**) Quantified growth data of the results shown in (**E**). (**G**) Western blot showing the protein expression levels of BRAF in indicated cell lines which were used in the assays shown in (**E,F**). Original western blots are presented in Supplemental Fig. S1D. For the bar graphs in (**B,F**)*,* showing the quantified data of the growth assays, the bars represent the average data from at least three independent experiments ± SEM. p-values are indicated with ***p < 0.001, **p < 0.01 and *p < 0.05 (two-tailed *t*-test).

The AR inhibitor enzalutamide is successfully used for the treatment of prostate cancer. However, primary resistance is observed in a significant proportion of patients^3,12^, illustrating the need for improved therapeutic approaches for this subset of patients. To address this unmet clinical need, we performed a kinome-centered CRISPR-Cas9 screen to identify kinases whose inhibition synergize with AR inhibition in cells that show a poor response to enzalutamide. The cell line CWR-R1 is a prostate cancer cell line that shows moderate sensitivity to the AR inhibitor enzalutamide, as compared to the sensitive LNCaP cells and resistant 22rv1 cells (Fig. 1A,B). This moderate sensitivity makes the CWR-R1 cell line a suitable model system to screen for kinases whose inhibition may synergize with enzalutamide to enhance anti-tumor effects in vitro.

CWR-R1 cells were infected with the NKI Human Kinome CRISPR pooled sgRNA library targeting 578 human kinases. Infected cells were seeded at low density and treated with 10 μM enzalutamide or vehicle for 2 weeks to allow selection. The single gRNA (sgRNA) cassettes were recovered from the genomic DNA by PCR and their relative abundance was determined through massively parallel sequencing (Fig. 1C). The sgRNA abundance of the enzalutamide-treated and vehicle-treated populations were compared, and depleted sgRNAs were identified using DESeq2^15^ and MAGeCK^16^ analyses (Supplemental Tables S1, S2). We found that all 10 sgRNAs targeting *BRAF* were under-represented in enzalutamide-treated cells when compared to vehicle-treated cells (Fig. 1D).

We validated the results of the CRISPR-Cas9 screen using sgRNAs targeting *BRAF* in CWR-R1 cells, using polyclonal knockout cell populations. Knockout of *BRAF* using two independent sgRNAs (sg*BRAF*-9 and sg*BRAF*10) yielded viable cells with growth kinetics mirroring those of control cells harboring a non-targeting sgRNA (sgNT) (Fig. 1E,F). CRISPR-mediated loss of BRAF protein expression in *BRAF*^KO^ cells was confirmed by western blot (Fig. 1G). Importantly, *BRAF*^KO^ cells showed increased sensitivity to enzalutamide in long-term growth assays when compared to control cells (Fig. 1E,F), concordant with the results from the screen. In contrast to our findings in CWR-R1 cells, knockout of *BRAF* using CRISPR-Cas9 in LNCaP cells did not result in increased sensitivity to enzalutamide (Supplemental Fig. S1A–C).

### MAPK inhibition potentiates enzalutamide sensitivity in AR-driven prostate cancer cells harboring a *BRAF* mutation

Given the increased sensitivity to enzalutamide upon knockout of *BRAF*, we sequenced the *BRAF* kinase domain in CWR-R1 cells using a clinically validated, NGS-based, targeted sequencing assay. Through this approach, we identified a p.L597R mutation in the activating kinase domain of the *BRAF* gene. Sequencing of LNCaP cells using the same assay, revealed no *BRAF* alterations, consistent with previous reports for this cell line^2,17^.

**Figure 2:**
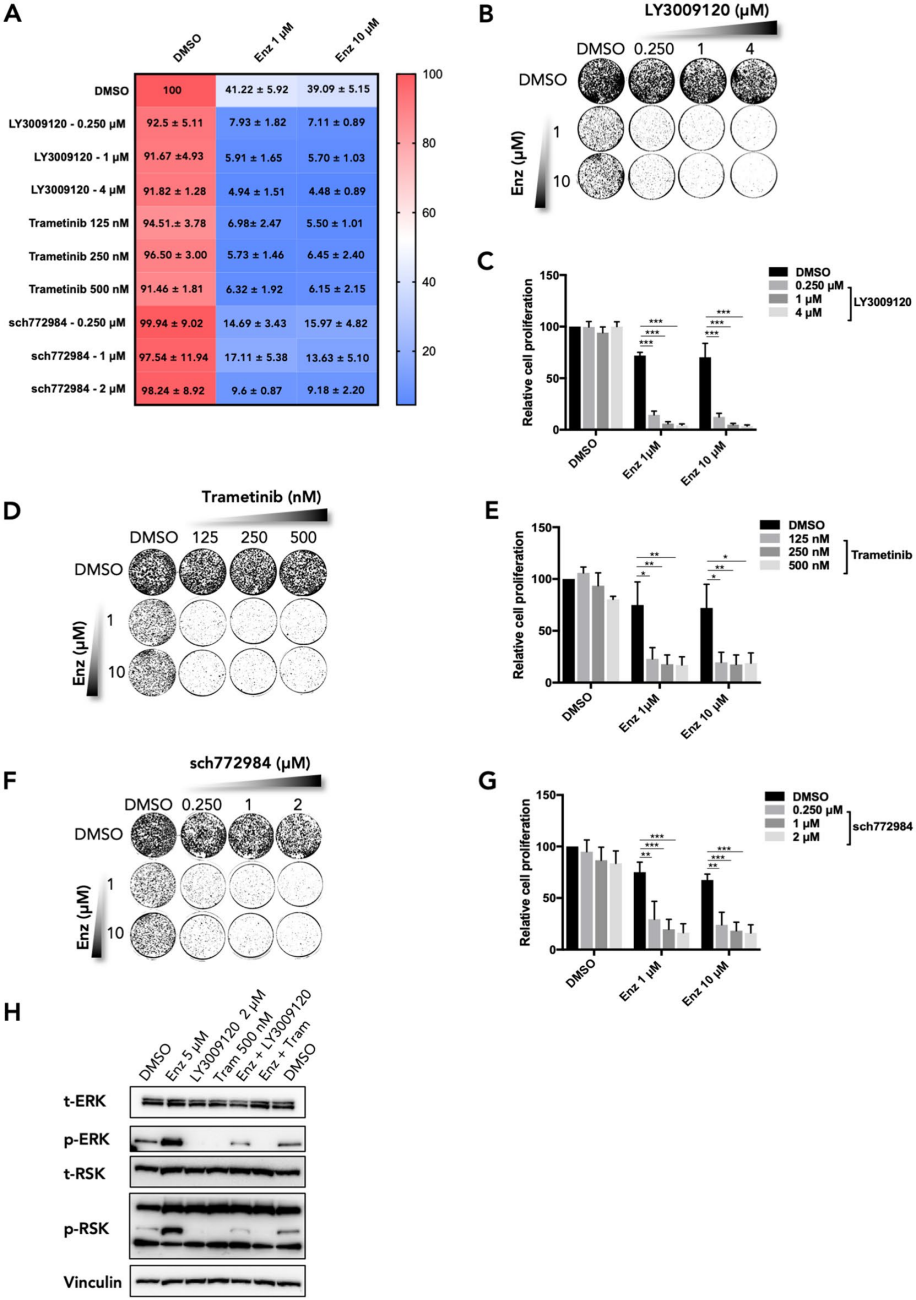
Pharmacological validation of screen hit *BRAF* in CWR-R1 prostate cancer cells. (**A**) Short-term growth assay for CWR-R1 cells treated with enzalutamide and MAPK pathway inhibitors. The percentage of growth relative to the untreated control is shown, with the standard error of the mean (SEM) for n = 3 experimental replicates. (**B,C**) Long-term growth assay of CWR-R1 cells treated with AR inhibitor enzalutamide and RAF inhibitor LY3009120 as monotherapy or in combination. (**D,E**) Long-term growth assay of CWR-R1 cells cultured in the presence of enzalutamide and MEK inhibitor trametinib as indicated. (**F,G**) Long-term growth assay of CWR-R1 cells treated with enzalutamide in the presence of ERK inhibitor sch772984 at indicated concentrations. (**H**) Western blot showing the expression levels and phosphorylation status of MAPK pathway components in CWR-R1 cells treated with inhibitors as indicated. Vinculin was used as a loading control. Original western blots are shown in Supplemental Fig. S6A. For the bar graphs showing the quantified data of the growth assays, the bars represent the average data from at least three independent experiments ± SEM. p-values are indicated with ***p < 0.001, **p < 0.01 and *p < 0.05 (two-tailed *t*-test).

Next, we assessed whether the increased sensitivity of *BRAF*^KO^ CWR-R1 cells to enzalutamide could be confirmed by pharmacological inhibition of the MAPK pathway in combination with enzalutamide. Short-term and long-term growth assays showed that CWR-R1 cells were unresponsive to the RAF inhibitor LY3009120 (Fig. 2A–C). However, the combination of LY3009120 and enzalutamide resulted in strong inhibition of cell proliferation when compared to monotherapy treatment using these two inhibitors (Fig. 2A–C). The combination of enzalutamide and dabrafenib was also tested and found to be more effective than single drug treatments (Supplemental Fig. S2A,B), but to a lesser extent when compared to the combination of enzalutamide and LY3009120. These findings are consistent with the reduced efficacy of BRAF V600E-targeting drugs, such as dabrafenib, in non-V600E *BRAF* mutant cancer cells^18^.

Pharmacological inhibition of the MAPK pathway downstream of BRAF was performed using trametinib, a MEK inhibitor, and sch772984, an ERK inhibitor. Trametinib monotherapy did not affect cell proliferation in short-term and long-term growth assays with concentrations up to 500 nM. However, when combined with enzalutamide, a strong inhibitory effect on growth was observed (Fig. 2A,D,E). Similar results were obtained with the ERK inhibitor sch772984, which showed a strong synergistic effect only when used in combination with enzalutamide (Fig. 2A,F,G). Growth assays using low concentrations of enzalutamide showed that co-treatment with MAPK inhibitors enhanced enzalutamide efficacy, making the drug effective in the nanomolar (nM) range, though the effect was strongest at 0.5 μM (Supplemental Fig. S2C–H).

Although LNCaP cells do not contain an activating *BRAF* mutation, other oncogenic alterations may cause MAPK pathway activation in these cells. However, inhibition of AR in combination with MEK or ERK inhibitors in this cell line did not result in increased sensitivity to enzalutamide (Supplemental Fig. S3A–D). These result are concordant with the absence of activating MAPK alterations^2,17^. LNCaP cells are *PTEN*-deficient and were shown to be more dependent on PI3K signaling upon enzalutamide-mediated AR inhibition, through reciprocal feedback regulation of the AR and PI3K pathway. It was shown that co-targeting of the PI3K and AR pathway in LNCaP cells resulted in strong anti-tumor effects when compared to single drug treatment^8^.

We also tested the combination of AR and RAF/MAPK inhibitors in two enzalutamide-resistant cell lines: AR-negative PC3 cells and the 22rv1 cell line. In 22rv1 cells, enzalutamide resistance was shown to be mediated by the AR-V7 splice variant^19^. Both cell lines did not respond to enzalutamide, as expected. Addition of inhibitors targeting MAPK pathway components RAF, MEK and ERK did not affect the enzalutamide response of PC3 cells (Supplemental Fig. S4A–C), excluding off-target effects of the used inhibitors. We found that 22rv1 cells showed modest sensitivity to MAPK pathway inhibitors (Supplemental Fig. S5A–C). MAPK inhibitor treatment had no effect on the enzalutamide response (Supplemental Fig. S5A–C), and knockout of *BRAF* did not confer enzalutamide sensitivity in these cells (Supplemental Fig. S5D–F). The lack of synergy between AR and MAPK inhibitors is most likely caused by the fact that enzalutamide is incapable of targeting the AR-V7 splice variant that drives resistance in 22rv1 cells, as AR-V7 lacks the ligand-binding domain.

Next, we biochemically investigated MAPK pathway activation in response to AR and MAPK inhibition as monotherapy and when used in combination in CWR-R1 cells. We found that upon enzalutamide treatment, MAPK signaling is upregulated, as shown by increased p-ERK and p-RSK levels (Fig. 2H)^20^. Inhibition of the MAPK pathway by LY3009120 or trametinib significantly downregulated p-ERK and p-RSK levels. When cells were treated with enzalutamide in addition to LY3009120, MAPK signaling was slightly increased, but still significantly lower when compared to enzalutamide monotherapy (Fig. 2H). This upregulation as a result of enzalutamide treatment was not observed when cells were treated with the combination of enzalutamide and trametinib (Fig. 2H). Together, these data suggest that MAPK inhibition in combination with enzalutamide may be effective in AR-driven prostate cancer cells having an activated MAPK pathway, through an activating *BRAF* mutation.

### Clinical response to enzalutamide in *BRAF*-mutant CRPC patients

**Figure 3:**
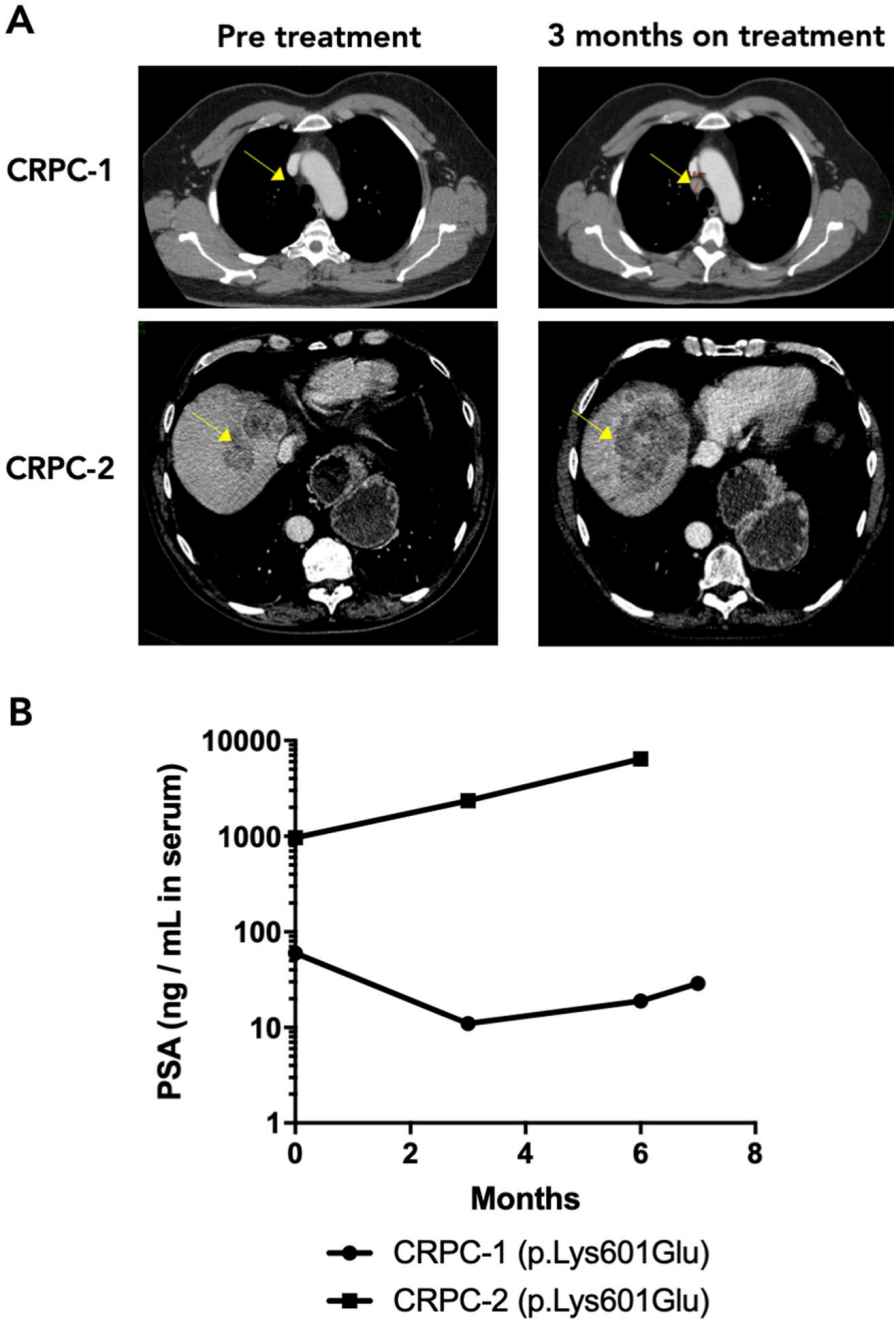
Enzalutamide response in two patients with *BRAF* mutant tumors. (**A**) Computed tomography (CT) imaging for two patients with tumors harboring the *BRAF* p.K601E mutation prior to starting treatment (left) and 3 months on treatment (right). The yellow arrow indicates a mediastinal lymph node in CRPC-1 and liver metastases in CRPC-2. (**B**) On-treatment PSA levels for the two patients shown in (**A**).

Although infrequent, prostate cancers harbor *BRAF* mutations in around 2% of cases (Supplemental Fig. S7A)^21,22^, mostly involving hotspot mutations p.K601E and p.G469A (Supplemental Fig. S7B). To explore the role of *BRAF* mutations in primary resistance to enzalutamide in CRPC patients, we analyzed sequencing data from a biopsy study (CPCT-02) at our center to identify *BRAF*-mutated patients. Biopsies were collected prior to enzalutamide or abiraterone treatment, and sequenced by either exome or whole genome sequencing^23^. We identified two patients harboring a *BRAF* p.K601E mutation. Both of these patients showed early clinical progression after commencing enzalutamide treatment (Fig. 3A,B). Out of the 30 similarly treated patients in the cohort, having clinical and PSA data available, 77% (n = 23) showed a decline of ≥ 50% in PSA levels in the first 3 months of enzalutamide or abiraterone treatment. After 6 months of treatment, 83% (n = 25) of patients had a lower PSA level compared to baseline (Supplemental Fig. S7C). These numbers are concordant with previously reported rates of primary resistance in enzalutamide-treated patients^3,12^. Our findings suggest that activating mutations in *BRAF* are associated with primary resistance to enzalutamide, though confirmation in a larger cohort of *BRAF*-mutated prostate cancer patients is needed.

Together, we find that co-inhibition of the AR and MAPK pathway activity is synergistic in prostate cancer cells carrying a *BRAF* mutation. Furthermore, the poor clinical response to enzalutamide in two CRPC patients harboring *BRAF* mutations indicates that oncogenic mutations in the kinase domain of *BRAF* may result in primary resistance to therapy, which could be addressed by co-treatment with MAPK inhibitors. These findings warrant further investigation of *BRAF* alterations in the context of the enzalutamide response in larger cohorts, to further elucidate the clinical relevance of activating *BRAF* mutations in AR inhibitor-treated patients.

## Discussion

AR antagonists, such as enzalutamide, are effective in the treatment of AR-driven prostate cancer^3^. Still, resistance to AR inhibitors commonly arises during therapy, and primary resistance occurs in 10–20% of patients^3,12^. To improve treatment outcome, more insight into the primary resistance mechanisms is essential and may lead to the development of new treatment avenues.

In our study, we employed a kinome-centered CRISPR-Cas9 screen to identify genes that can be targeted to improve sensitivity to AR inhibition. We found that genetic knockout of the *BRAF* gene resulted in increased sensitivity to enzalutamide in CWR-R1 cells. These findings were confirmed through pharmacological inhibition of BRAF, or downstream components of the MAPK pathway. Through genetic profiling of CWR-R1 cells, the *BRAF* p.L597R mutation was identified, potentially conferring specific vulnerability to BRAF inhibition in CWR-R1 cells. The clinical significance of *BRAF* p.L597R has been shown in melanoma patients, where expression of this mutant was associated with sensitivity to MEK inhibitors^24,25^. Moreover, knockdown experiments comparing WT *BRAF* to several mutant forms of *BRAF*, including p.L597R, demonstrated the oncogenic function of this *BRAF* mutant in non-small cell lung cancer (NSCLC) cells^26^. In CWR-R1 cells, increased BRAF activity as a result of this mutation may be responsible for the moderate sensitivity of these cells to enzalutamide, and their sensitivity to MAPK inhibition in combination with AR blockade. The fact that no mutations affecting *BRAF* are present in LNCaP^2,17^, may explain the lack of synergy of combined AR/MAPK inhibition in this cell line.

Driver mutations in *BRAF* are found in a variety of cancers and are characterized by activating hotspot mutations in the kinase domain of the gene, most notably the p.V600E mutation^27,28^. Cancers harboring *BRAF* mutations are often sensitive to BRAF inhibitors, such as dabrafenib or vemurafenib^28^. Sensitivity can be increased by combination with MEK inhibitors, such as trametinib^29^. *BRAF* mutations in prostate cancer are rare but do occur in around 2% of patients^21,22^, predominantly involving hotspot mutations in the activating kinase domain (p.K601E and p.G469A; Supplemental Fig. S7A,B)^21,22,30^. In our study, we describe two mCRPC patients with tumors harboring a p.K601E *BRAF* mutation with early disease progression after commencing enzalutamide treatment, indicating potential relevance of these mutations in driving prostate cancer cell growth and enzalutamide resistance. Validation of these findings in larger cohorts is needed to confirm whether presence of these mutations correlates with primary resistance in patients. Our findings suggest that co-inhibition of AR and BRAF in *BRAF*-mutant prostate cancer patients could be particularly effective.

Alterations in the MAPK pathway are observed in about 40% of primary and 90% of metastatic prostate cancer cases^2^. Amplification of MAPK components is frequent, while mutations in members of this pathway are less common in prostate cancer^2,31^. Whereas the clinical significance of *BRAF* mutations has been demonstrated in various disease settings, it is unclear what proportion of the MAPK alterations found in prostate cancer lead to meaningful activation of the MAPK pathway. Emerging evidence suggests that targeting the MAPK pathway may represent a viable treatment approach for advanced prostate cancer cells fully resistant to enzalutamide^20,31^.

In conclusion, the findings by our group and others warrant further investigation of combined inhibition of the MAPK and AR pathway at an early stage of systemic treatment of AR-driven prostate cancer to overcome primary or acquired resistance. However, investigation of the clinical progression and longitudinal biochemical responses of enzalutamide-treated patients in larger cohorts is needed to further validate our findings in a clinical setting. The increase in availability of genetic profiling for cancer mutations in clinical settings^32^ may aid further exploration of the potential for combined BRAF/AR inhibition in *BRAF*-mutant prostate cancer.

## Materials and methods

### Cell culture and generation of knockout cells

The human prostate cancer cell lines LNCaP, CWR-R1, 22rv1 and PC3 were a kind gift from Prof. W. Zwart (Netherlands Cancer Institute). All prostate cancer cell lines were maintained in RPMI. HEK293T cells were obtained from ATCC and were cultured in DMEM. Medium was supplemented with 10% FBS (Serana) and 1% penicillin/streptomycin. Cells were maintained at 37 °C in 5% CO_2_. All cell lines were STR profiled. Control and *BRAF*^KO^ cells were created by infecting target cells with lentiviral particles containing LentiCRISPR v2.0 harboring non-targeting or *BRAF*-targeting gRNAs, which were cloned into the vector using Gibson Assembly (NEB cat#: E2611S) utilizing BsmBI restriction sites. For gRNA sequences see Supplemental Table S3. For virus production, HEK293T cells were co-transfected with lentiviral CRISPR constructs, using PEI. Target cells were seeded 1 day prior to infection. Lentiviral supernatant was added to the medium along with 5 μg/ml polybrene. Infected cells were selected with 2 μg/ml puromycin.

### Kinome-centered CRISPR-Cas9 dropout screen

CWR-R1 cells were infected with lentiviral particles containing the NKI Human Kinome CRISPR Knockout library at low M.O.I. (~ 0.2) for single viral integration, at a ~ 500-fold coverage, and cultured in the presence of vehicle or 10 μM enzalutamide for ~ 2 weeks. Barcodes were recovered and sequenced as described^11^. For sequence depth normalization a relative total size factor was calculated for each sample, by dividing the total counts of each sample by the geometric mean of all totals. After normalization, a differential test between the treated and untreated condition for each sgRNA was performed using DESeq2^15^. The output from the DESeq2 analysis contains the DESeq2 test statistic. Positive DESeq2 test statistic indicate positive log2FoldChange value, negative DESeq2 test statistic indicate negative log2FoldChange value. We sorted the output of DESeq2 on the test statistic in increasing order, putting the most significant depleted sgRNA at the top. We then used the MAGeCK^16^ Robust Rank Algorithm to determine for each gene if its sgRNAs are enriched towards the top of the result list. The resulting enrichment p-values were corrected for multiple testing using the Benjamini–Hochberg correction, resulting in a FDR value. As hits we considered the genes with a FDR rounded on two decimals ≤ 0.1.

### Proliferation assays

Colony formation assays were performed as previously described^11^. Enzalutamide, LY3009120, Dabrafenib, Trametinib, sch772984 were obtained from Medkoo Biosciences, all drugs were dissolved in DMSO and stored at − 20 °C. Used seeding densities were 20,000 (LNCaP) or 10,000 (22rv1, CWR-R1) cells/well in 6-well plates, and drugs were added as indicated the next day. For 12-well assays, the used seeding densities were 10,000 (LNCaP), or 5000 (CWR-R1, 22rv1, PC3). The growth medium, containing vehicle or drugs, was refreshed every 36–48 h. After 12–14 days of growth in presence of the drugs, when the control cells reached ~ 90% confluency, all cells were fixed in 2% formaldehyde and stained with 0.1% crystal violet.

For quantification of the growth assays, crystal violet was extracted by incubating the stained plates with 5% acetic acid for 1 h at room temperature. The solution, containing the crystal violet, was transferred to a 96-well plate and measured using the Envision 2104 Multilabel Reader (PerkinElmer). Growth assays were performed at least three times for each experiment. Therefore, when quantified data is shown, bars represent the average data from at least three independent experiments ± SEM. P-values are indicated with ***p < 0.001, **p < 0.01 and *p < 0.05 (two-tailed *t*-test).

### Protein lysate preparation and western blot

Typically, CWR-R1 cells were plated at a density of 200,000 cells per well in 6-well plates and cultured in the presence of drugs as indicated for 5 days before harvesting. Samples were prepared and western blot was performed as described previously^11^, using the spectra Multicolor Broad Range Protein Ladder (ThermoFisher Scientific). Antibodies directed against BRAF (14814), GAPDH (5174), t-ERK (9102), p-ERK (4377), t-RSK (8408) were purchased from Cell Signaling Technology; antibody against p-RSK (04-419) was purchased from Millipore; antibody targeting Vinculin (V9131) was purchased from Sigma.

## Supplementary Information

Supplementary Table S1.

Supplementary Table S2.

Supplementary Table S3.

Supplementary Figures.
