# Supplementary material for: A kinome-centered CRISPR-Cas9 screen identifies activated BRAF to modulate enzalutamide resistance with potential therapeutic implications in BRAF-mutated prostate cancer
Source: Sci Rep. 2021 Jul 1;11:13683. doi: 10.1038/s41598-021-93107-w (PMC8249522; doi:10.1038/s41598-021-93107-w)
Supplement: Supplementary file 3 — Supplementary Table S3. [file 41598_2021_93107_MOESM3_ESM.docx]

**Supplemental Table S3:** Overview of sgRNA sequences

| **sgRNA** | **Sequence 5’-3’** |
| --- | --- |
| sgNT | ACGGAGGCTAAGCGTCGCAA |
| sgBRAF-9 | GGGCCAGGCTCTGTTCAACG |
| sgBRAF-10 | TATAAGATGGCGGCGCTGAG |
