# Supplementary material for: A kinome-centered CRISPR-Cas9 screen identifies activated BRAF to modulate enzalutamide resistance with potential therapeutic implications in BRAF-mutated prostate cancer
Source: Sci Rep. 2021 Jul 1;11:13683. doi: 10.1038/s41598-021-93107-w (PMC8249522; doi:10.1038/s41598-021-93107-w)
Supplement: Supplementary file 4 — Supplementary Figures. [file 41598_2021_93107_MOESM4_ESM.pdf]

A kinome-centered CRISPR-Cas9 screen identifies activated BRAF to modulate enzalutamide resistance with potential therapeutic implications in *BRAF*-mutated prostate cancer

Sander A. L. Palit[1*], Jeroen van Dorp[1,2], Daniel J. Vis[1], Cor Lieftink[3], Simon Linder[4,5], Roderick Beijersbergen[1,3], Andries M. Bergman[2,4], Wilbert Zwart[4,5] and Michiel S. van der Heijden[1,2*]

Supplementary information

Supplemental Figure S1

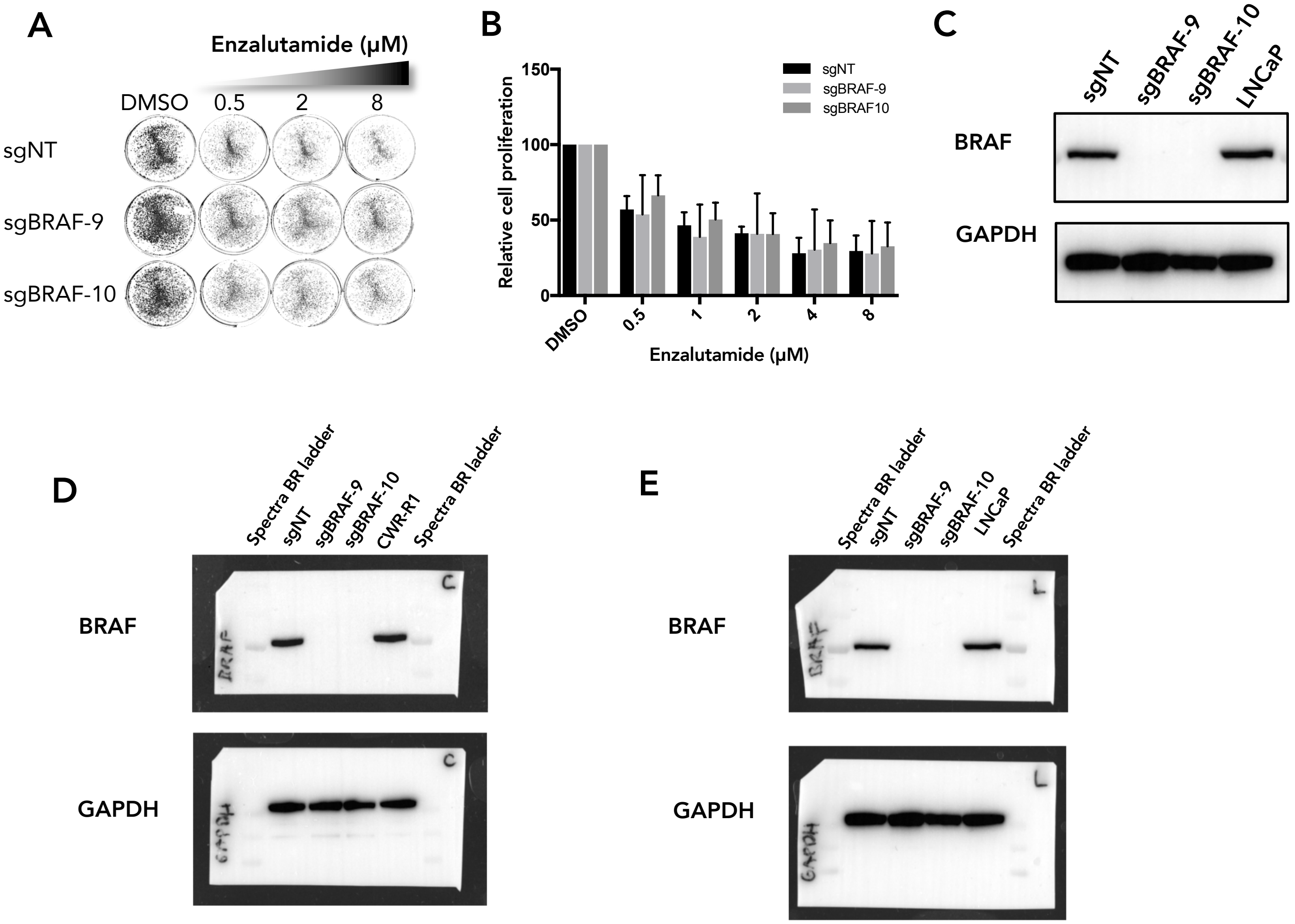

Supplemental Figure S1: (A) Long-term growth assay for LNCaP cells harboring control or *BRAF*-targeting sgRNAs, cultured in the presence of vehicle or enzalutamide as indicated. (B) Quantified data for the results shown in *A*. Bars represent the average data from at least three independent experiments, error bars represent SEM. (C) Western blot showing protein expression levels for BRAF and GAPDH in control and LNCaP $BRAF^{KO}$ cells. (D-E) Original western blot data for Fig. 1G and Supplemental Fig. 1C, respectively.

Supplemental Figure S2

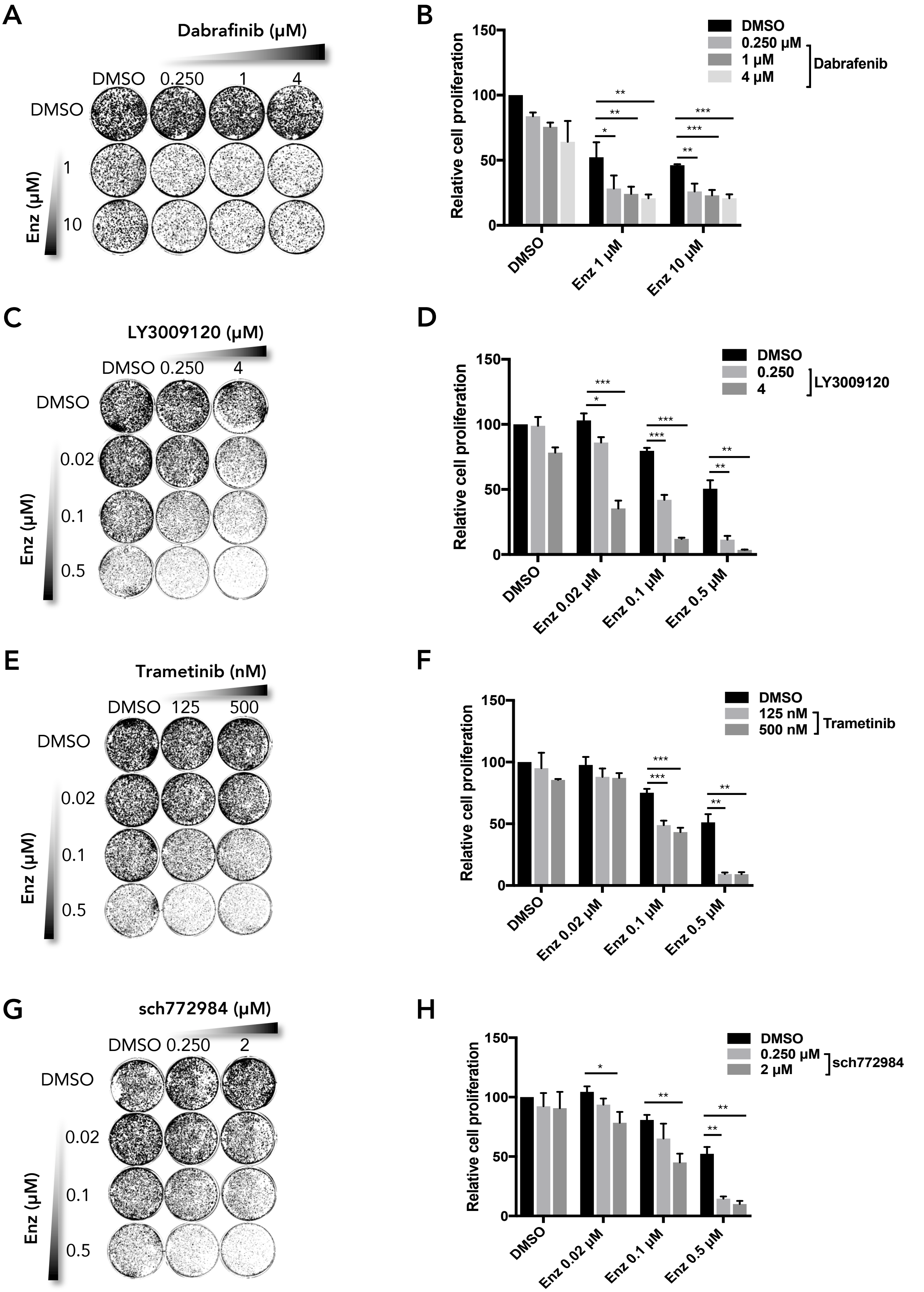

Supplemental Figure S2: (A-H) Long-term growth assays for CWR-R1 cells treated with indicated inhibitors are shown (left panels) together with quantification of these results (right panels) for n=3 experimental replicates. Bars represent the average data from at least three independent experiments, error bars represent SEM. P-values are indicated with ***p<0.001, **p<0.01 and *p<0.05 (two-tailed *t*-test).

Supplemental Figure S3

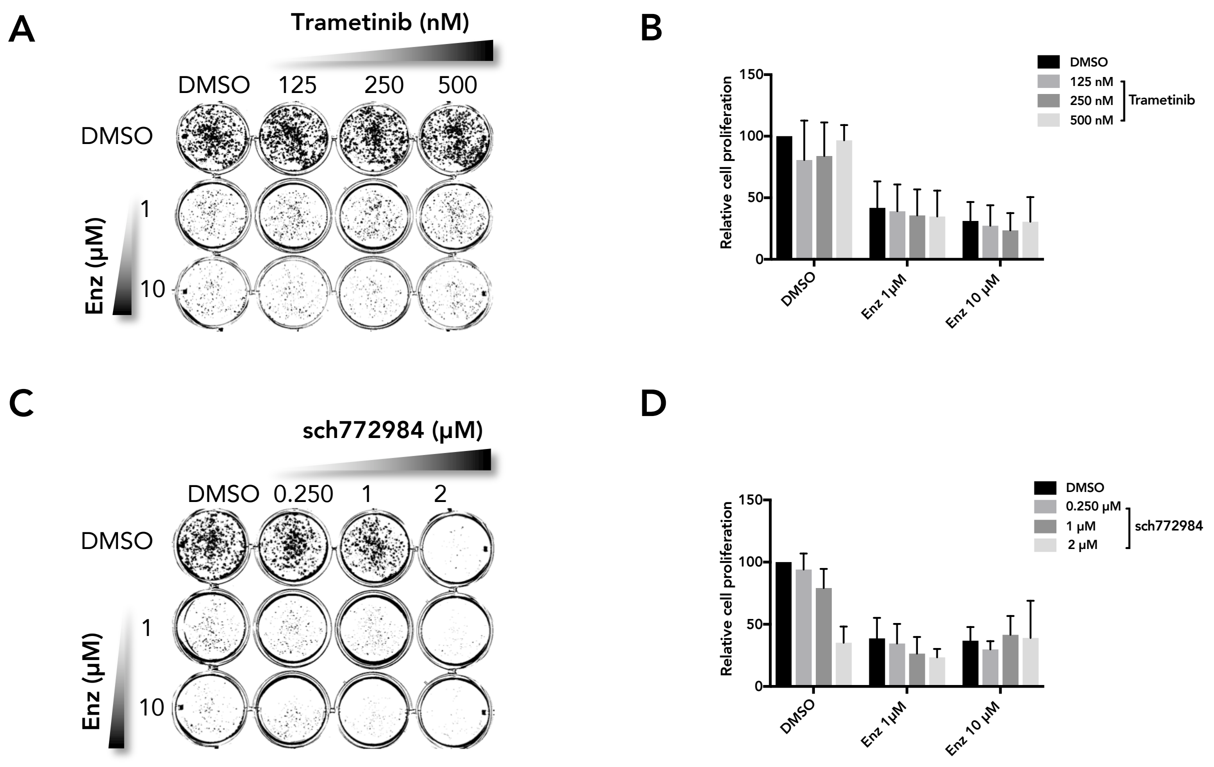

Supplemental Figure S3: (A-D) Long-term growth assays for LNCaP cells cultured in the presence of indicated inhibitors are shown (left panels), together with quantified results for n=3 experimental replicates (right panels). Bars represent the average data from at least three independent experiments, error bars represent SEM.

Supplemental Figure S4

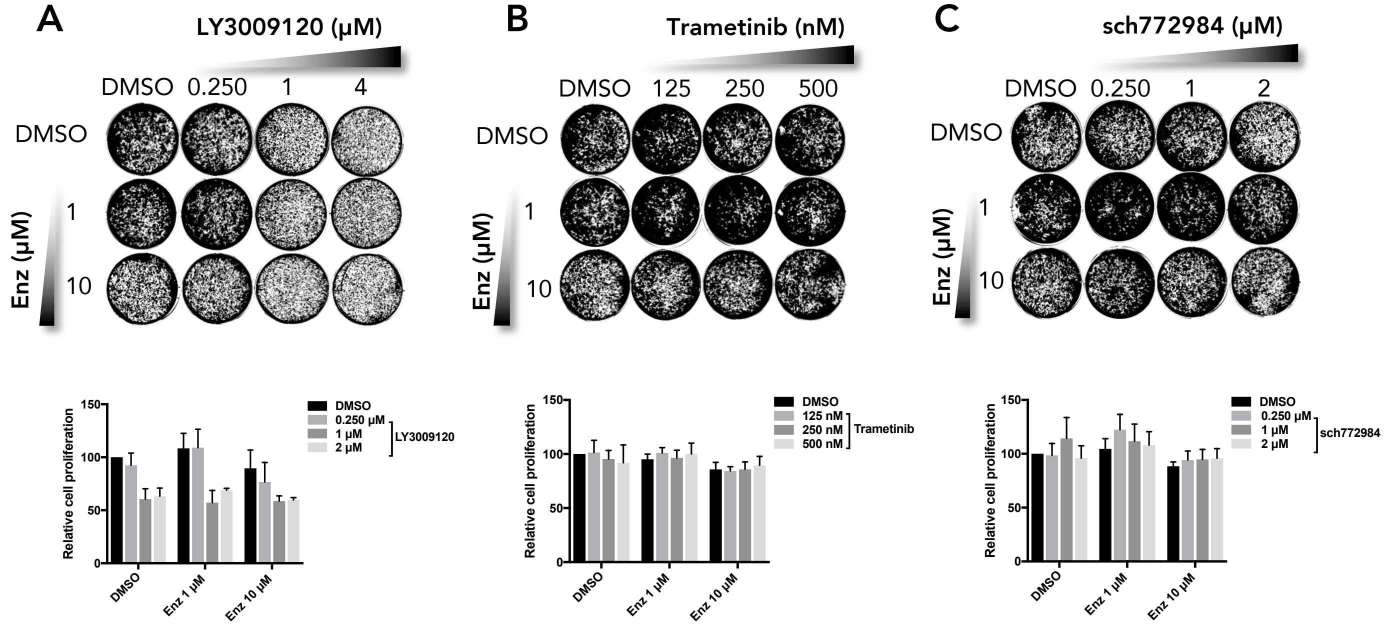

Supplemental Figure S4: (A-C) Long-term growth assays for PC3 cells treated with indicated inhibitors (top panels), together with the quantified results for n=3 experimental replicates (bottom panels). Bars represent the average data from at least three independent experiments, error bars represent SEM. P-values are indicated with ***p<0.001, **p<0.01 and *p<0.05 (two-tailed *t*-test).

Supplemental Figure S5

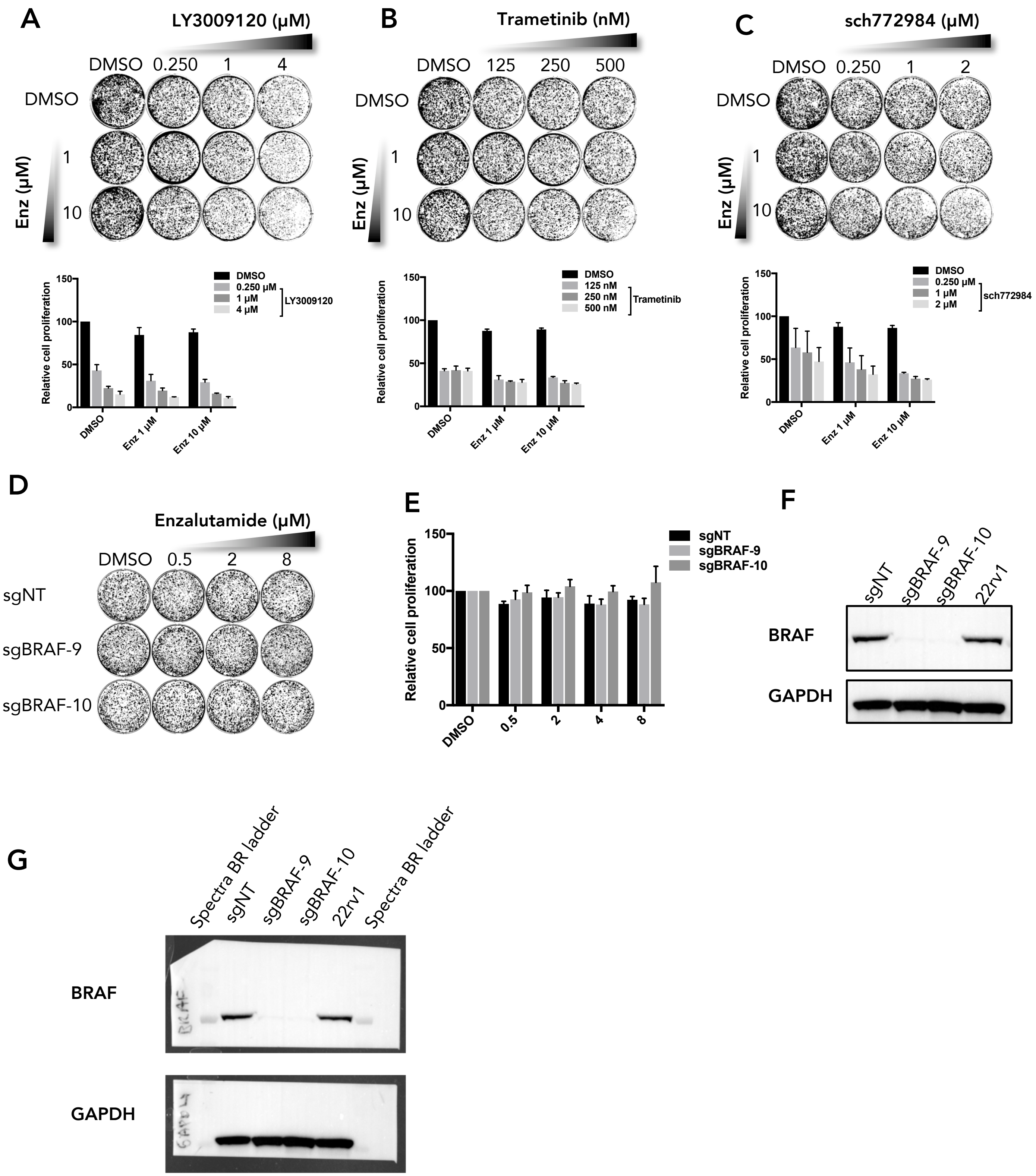

Supplemental Figure S5: (A-C) Long-term growth assays for 22rv1 cells treated with indicated inhibitors are shown (top panels), together with the quantified results (bottom panels). (D) Long-term growth assays for 22rv1 *BRAF*^{KO} cells treated with vehicle or enzalutamide at indicated concentrations. (E) Quantified data for the results shown in *D,* for n=3 experimental replicates. (F) Western blot showing the protein levels for BRAF in control and *BRAF*^{KO} 22rv1 cells, GAPDH was used as a loading control. (G) Original western blot data for Supplemental Fig. S5F. For the bar graphs in *A-C* and *E,* showing the quantified data of the growth assays, the bars represent the average data from at least three independent experiments with error bars showing the SEM.

Supplemental Figure S6

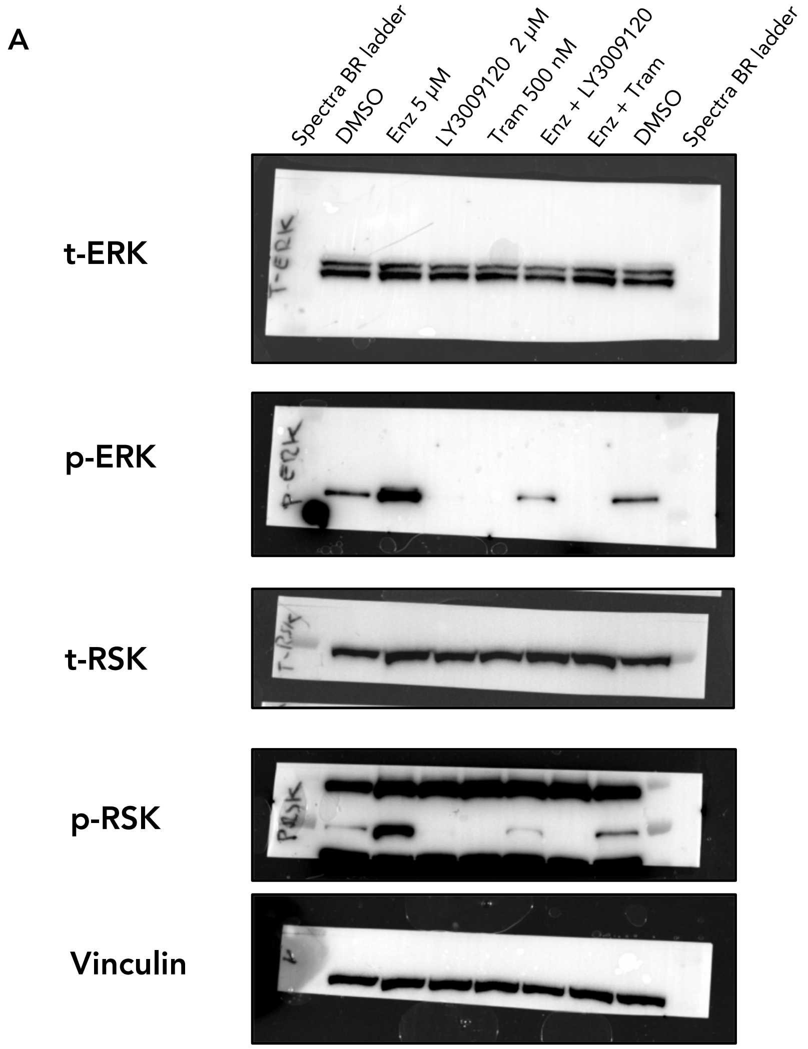

Supplemental Figure S6: (A) Original western blot data for Fig. 2H, showing the protein levels and phosphorylation status of MAPK components ERK and RSK in CWR-R1 cells cultured with drugs as indicated. Vinculin was used as a loading control.

Supplemental Figure S7

A

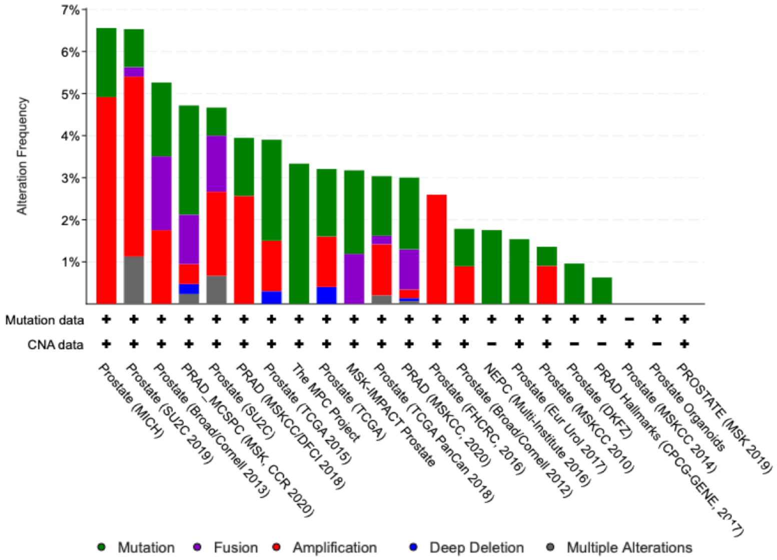

B

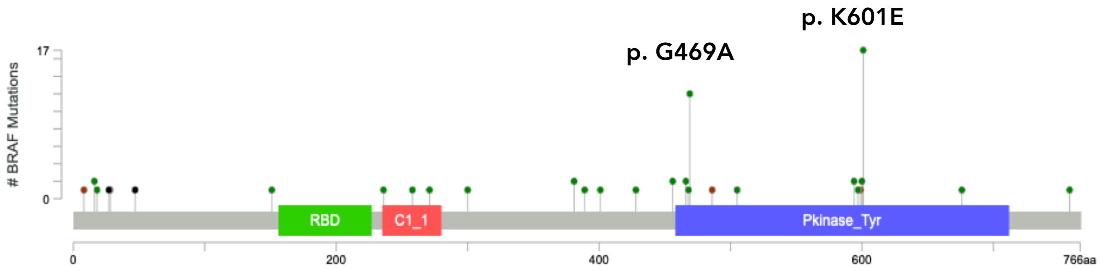

C

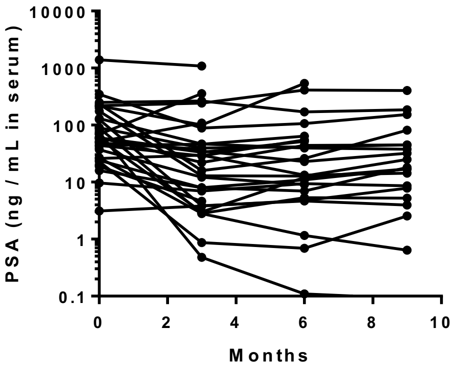

Supplemental Figure S7: (A) Overview of *BRAF* alterations in prostate cancer found in indicated studies[21,22]. (B) Overview of the frequency and nature of *BRAF* mutations in prostate cancer found in the studies indicated in *A*. (C) PSA response from enzalutamide-treated patients (n=30), harboring WT BRAF tumors, of the CPCT-02 cohort with available on-treatment PSA samples.
